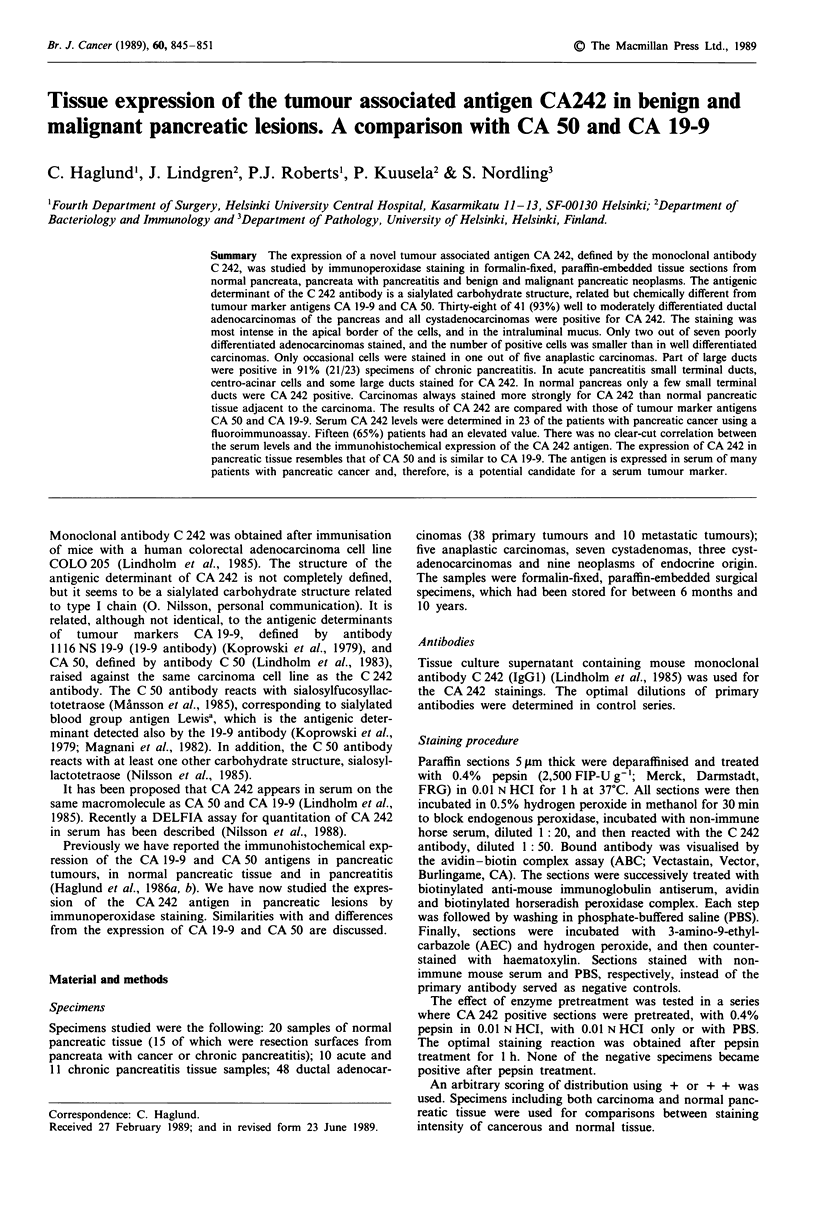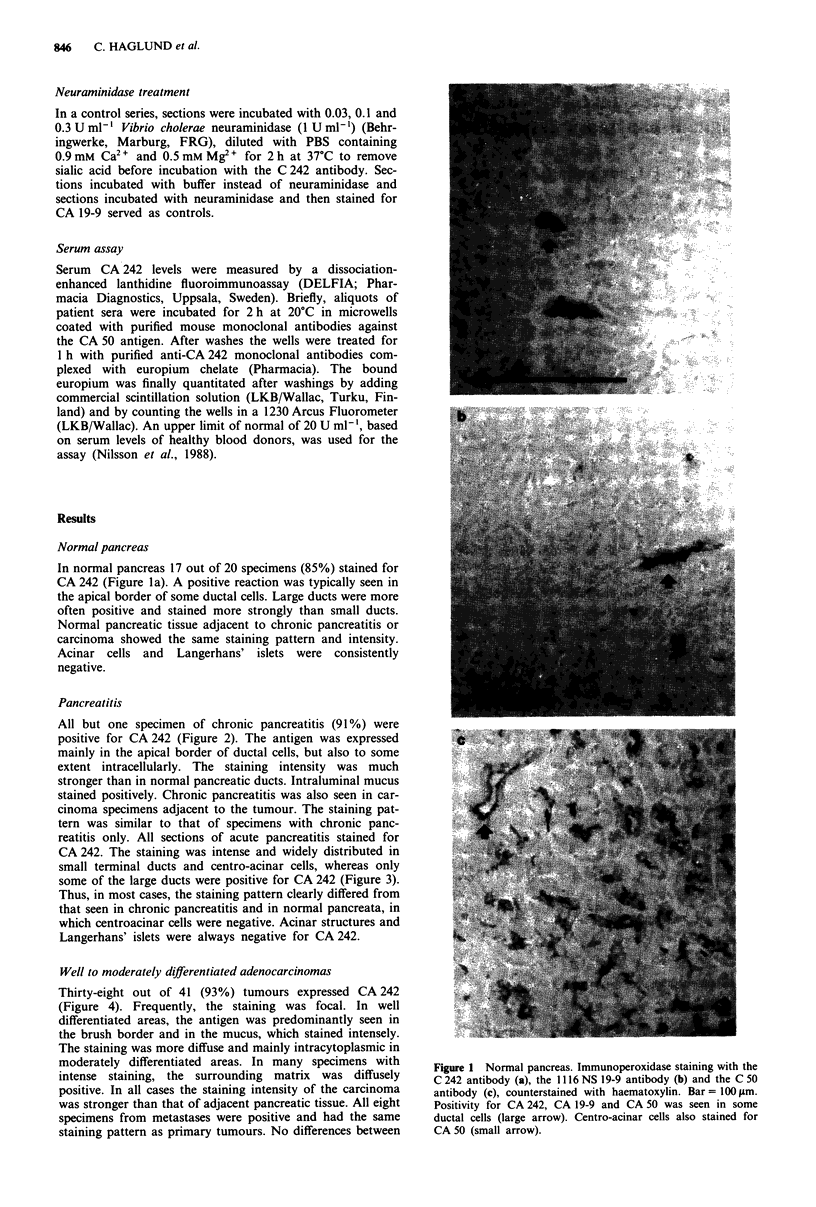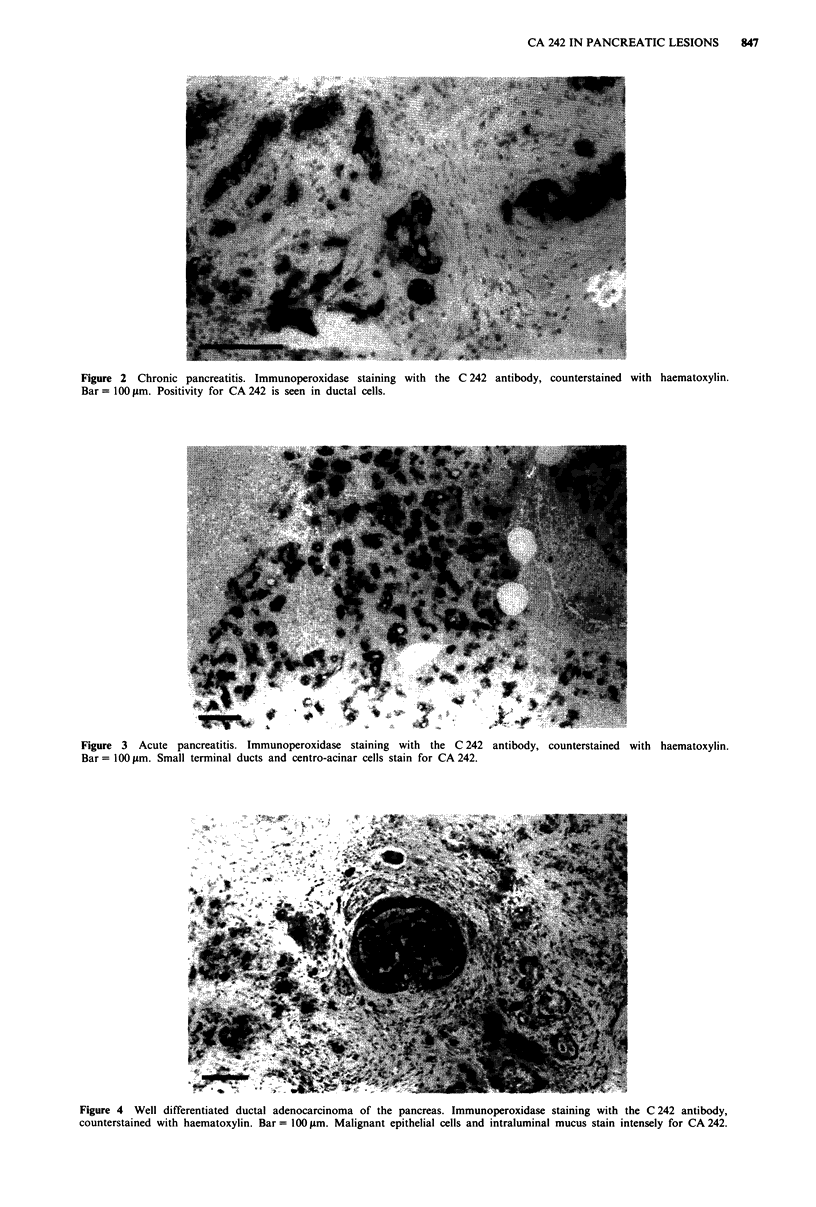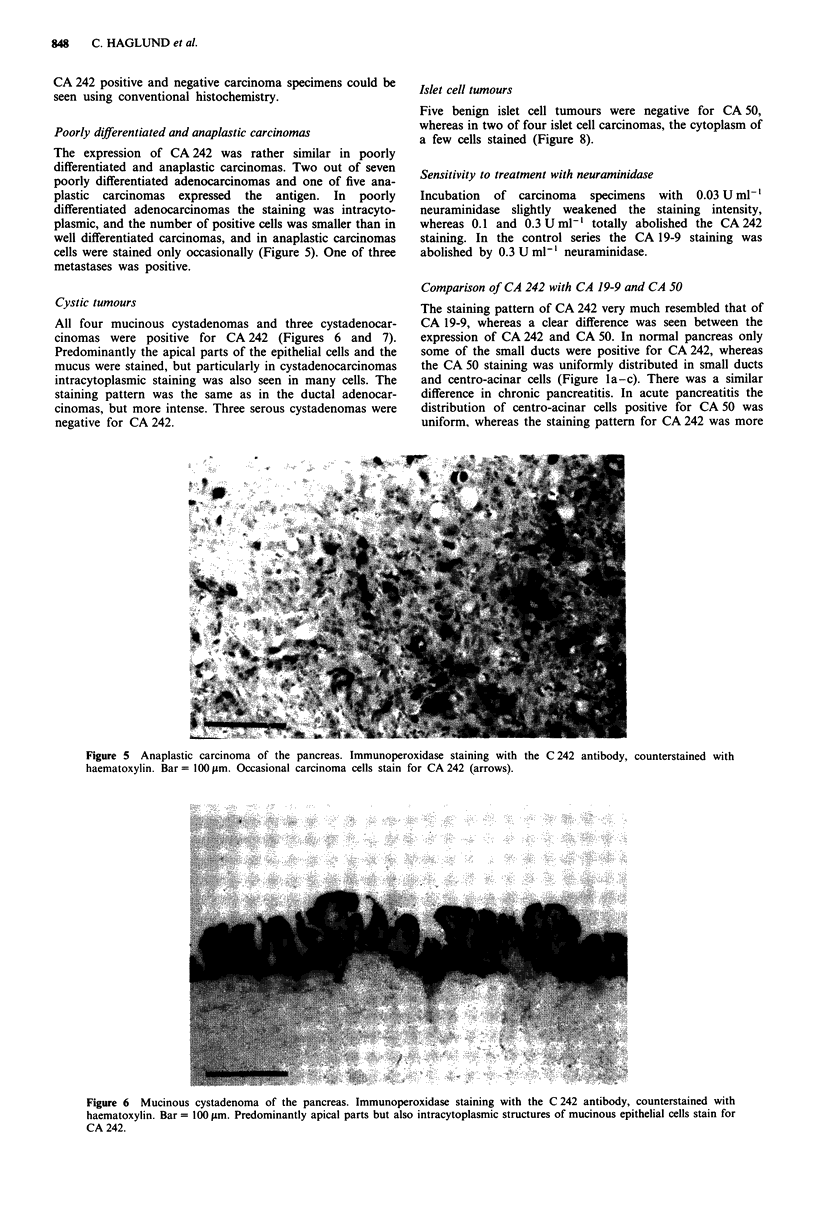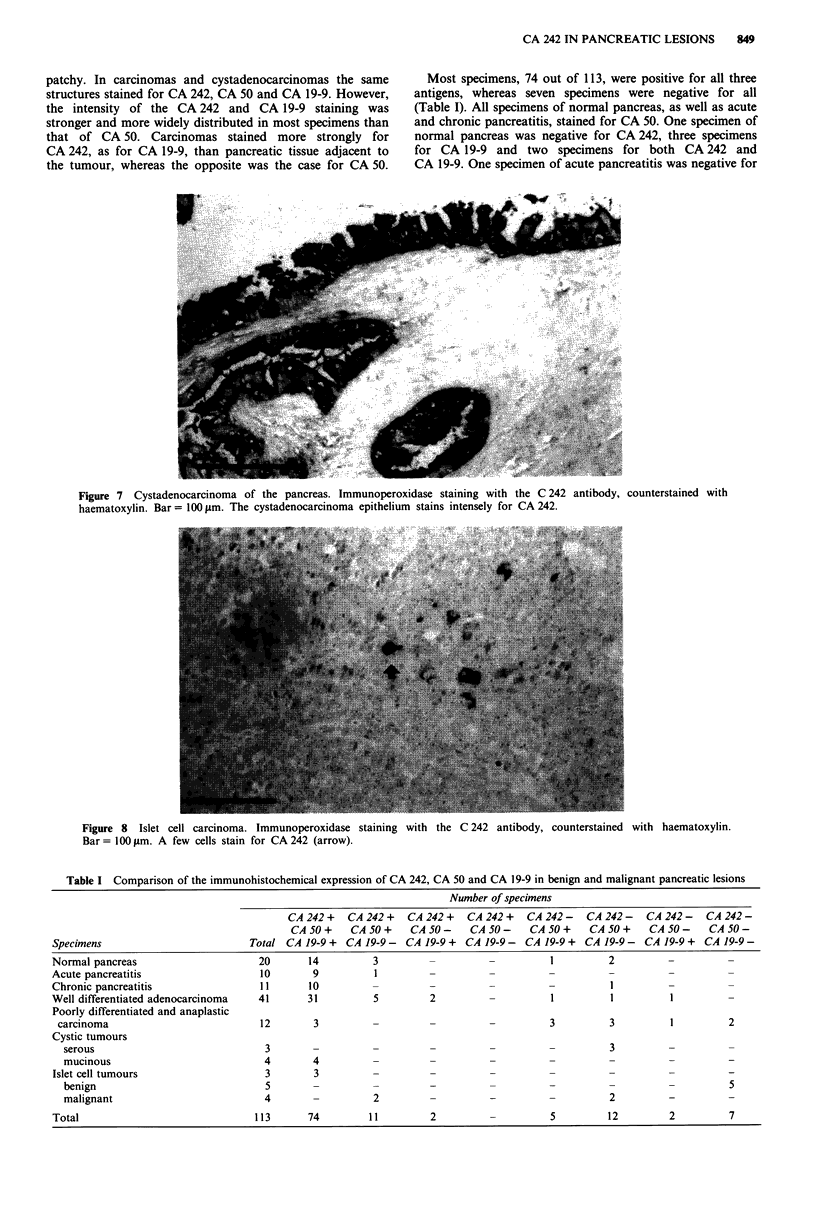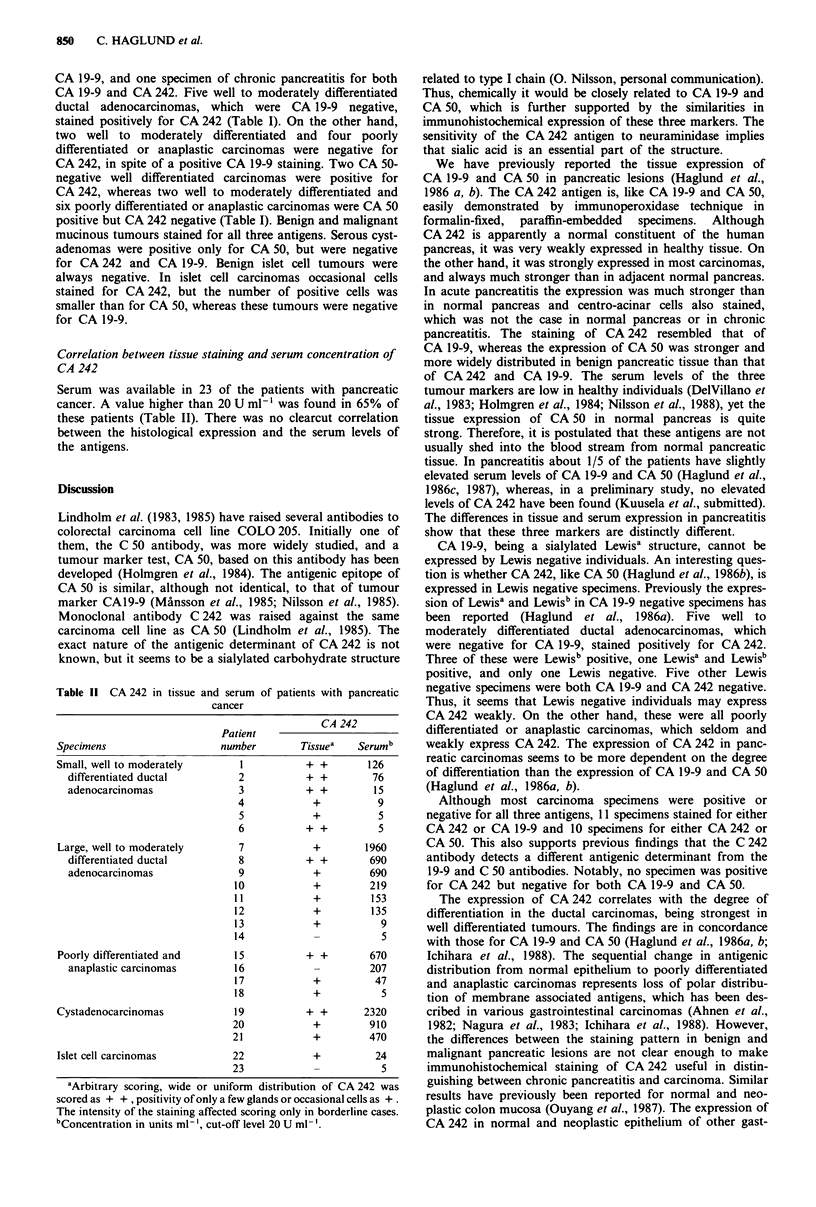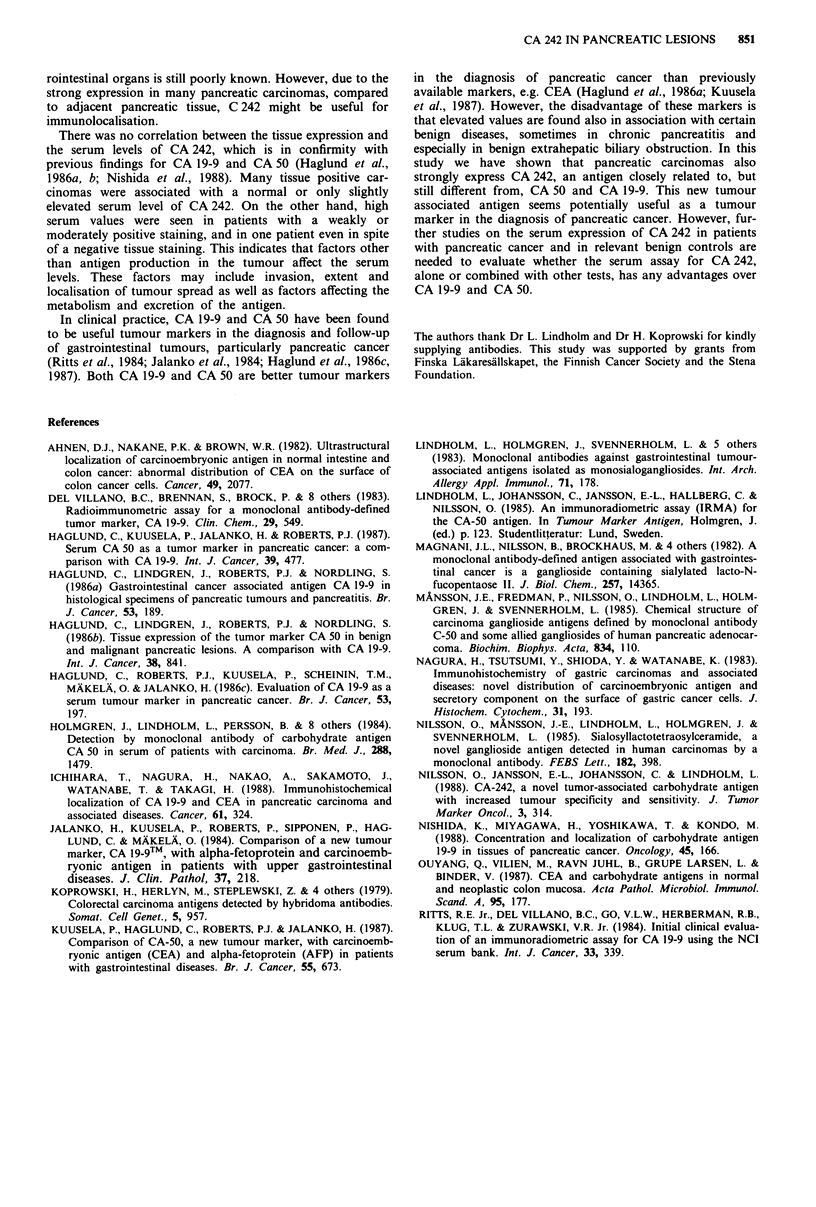# Tissue expression of the tumour associated antigen CA242 in benign and malignant pancreatic lesions. A comparison with CA 50 and CA 19-9.

**DOI:** 10.1038/bjc.1989.377

**Published:** 1989-12

**Authors:** C. Haglund, J. Lindgren, P. J. Roberts, P. Kuusela, S. Nordling

**Affiliations:** Fourth Department of Surgery, Helsinki University Central Hospital, Finland.

## Abstract

**Images:**


Br. J. Cancer (1989), 60, 845–851 © The Macmillan Press Ltd., 1989

### Tissue expression of the tumour associated antigen CA242 in benign and malignant pancreatic lesions. A comparison with CA 50 and CA 19-9

C. Haglund[1], J. Lindgren[2], P.J. Roberts[1], P. Kuusela[2] & S. Nordling[3]

[1]*Fourth Department of Surgery, Helsinki University Central Hospital, Kasarmikatu 11–13, SF-00130 Helsinki;* [2]*Department of Bacteriology and Immunology and* [3]*Department of Pathology, University of Helsinki, Helsinki, Finland.*

**Summary** The expression of a novel tumour associated antigen CA 242, defined by the monoclonal antibody C 242, was studied by immunoperoxidase staining in formalin-fixed, paraffin-embedded tissue sections from normal pancreata, pancreata with pancreatitis and benign and malignant pancreatic neoplasms. The antigenic determinant of the C 242 antibody is a sialylated carbohydrate structure, related but chemically different from tumour marker antigens CA 19-9 and CA 50. Thirty-eight of 41 (93%) well to moderately differentiated ductal adenocarcinomas of the pancreas and all cystadenocarcinomas were positive for CA 242. The staining was most intense in the apical border of the cells, and in the intraluminal mucus. Only two out of seven poorly differentiated adenocarcinomas stained, and the number of positive cells was smaller than in well differentiated carcinomas. Only occasional cells were stained in one out of five anaplastic carcinomas. Part of large ducts were positive in 91% (21/23) specimens of chronic pancreatitis. In acute pancreatitis small terminal ducts, centro-acinar cells and some large ducts stained for CA 242. In normal pancreas only a few small terminal ducts were CA 242 positive. Carcinomas always stained more strongly for CA 242 than normal pancreatic tissue adjacent to the carcinoma. The results of CA 242 are compared with those of tumour marker antigens CA 50 and CA 19-9. Serum CA 242 levels were determined in 23 of the patients with pancreatic cancer using a fluoroimmunoassay. Fifteen (65%) patients had an elevated value. There was no clear-cut correlation between the serum levels and the immunohistochemical expression of the CA 242 antigen. The expression of CA 242 in pancreatic tissue resembles that of CA 50 and is similar to CA 19-9. The antigen is expressed in serum of many patients with pancreatic cancer and, therefore, is a potential candidate for a serum tumour marker.

Monoclonal antibody C 242 was obtained after immunisation of mice with a human colorectal adenocarcinoma cell line COLO 205 (Lindholm *et al.*, 1985). The structure of the antigenic determinant of CA 242 is not completely defined, but it seems to be a sialylated carbohydrate structure related to type I chain (O. Nilsson, personal communication). It is related, although not identical, to the antigenic determinants of tumour markers CA 19-9, defined by antibody 1116 NS 19-9 (19-9 antibody) (Koprowski *et al.*, 1979), and CA 50, defined by antibody C 50 (Lindholm *et al.*, 1983), raised against the same carcinoma cell line as the C 242 antibody. The C 50 antibody reacts with sialosylfucosyllactotetraose (Månsson *et al.*, 1985), corresponding to sialylated blood group antigen Lewis[a], which is the antigenic determinant detected also by the 19-9 antibody (Koprowski *et al.*, 1979; Magnani *et al.*, 1982). In addition, the C 50 antibody reacts with at least one other carbohydrate structure, sialosyllactotetraose (Nilsson *et al.*, 1985).

It has been proposed that CA 242 appears in serum on the same macromolecule as CA 50 and CA 19-9 (Lindholm *et al.*, 1985). Recently a DELFIA assay for quantitation of CA 242 in serum has been described (Nilsson *et al.*, 1988).

Previously we have reported the immunohistochemical expression of the CA 19-9 and CA 50 antigens in pancreatic tumours, in normal pancreatic tissue and in pancreatitis (Haglund *et al.*, 1986*a*, *b*). We have now studied the expression of the CA 242 antigen in pancreatic lesions by immunoperoxidase staining. Similarities with and differences from the expression of CA 19-9 and CA 50 are discussed.

### Material and methods

### *Specimens*

Specimens studied were the following: 20 samples of normal pancreatic tissue (15 of which were resection surfaces from pancreata with cancer or chronic pancreatitis); 10 acute and 11 chronic pancreatitis tissue samples; 48 ductal adenocarcinomas (38 primary tumours and 10 metastatic tumours); five anaplastic carcinomas, seven cystadenomas, three cystadenocarcinomas and nine neoplasms of endocrine origin. The samples were formalin-fixed, paraffin-embedded surgical specimens, which had been stored for between 6 months and 10 years.

### *Antibodies*

Tissue culture supernatant containing mouse monoclonal antibody C 242 (IgG1) (Lindholm *et al.*, 1985) was used for the CA 242 stainings. The optimal dilutions of primary antibodies were determined in control series.

### *Staining procedure*

Paraffin sections 5 μm thick were deparaffinised and treated with 0.4% pepsin (2,500 FIP-U $g^{-1}$; Merck, Darmstadt, FRG) in 0.01 N HCl for 1 h at 37°C. All sections were then incubated in 0.5% hydrogen peroxide in methanol for 30 min to block endogenous peroxidase, incubated with non-immune horse serum, diluted 1 : 20, and then reacted with the C 242 antibody, diluted 1 : 50. Bound antibody was visualised by the avidin–biotin complex assay (ABC; Vectastain, Vector, Burlingame, CA). The sections were successively treated with biotinylated anti-mouse immunoglobulin antiserum, avidin and biotinylated horseradish peroxidase complex. Each step was followed by washing in phosphate-buffered saline (PBS). Finally, sections were incubated with 3-amino-9-ethylcarbazole (AEC) and hydrogen peroxide, and then counterstained with haematoxylin. Sections stained with non-immune mouse serum and PBS, respectively, instead of the primary antibody served as negative controls.

The effect of enzyme pretreatment was tested in a series where CA 242 positive sections were pretreated, with 0.4% pepsin in 0.01 N HCl, with 0.01 N HCl only or with PBS. The optimal staining reaction was obtained after pepsin treatment for 1 h. None of the negative specimens became positive after pepsin treatment.

An arbitrary scoring of distribution using + or + + was used. Specimens including both carcinoma and normal pancreatic tissue were used for comparisons between staining intensity of cancerous and normal tissue.

---

Correspondence: C. Haglund.
Received 27 February 1989; and in revised form 23 June 1989.

*Neuraminidase treatment*

In a control series, sections were incubated with 0.03, 0.1 and 0.3 U $ml^{-1}$ *Vibrio cholerae* neuraminidase (1 U $ml^{-1}$) (Behringwerke, Marburg, FRG), diluted with PBS containing 0.9 mM $Ca^{2+}$ and 0.5 mM $Mg^{2+}$ for 2 h at 37°C to remove sialic acid before incubation with the C 242 antibody. Sections incubated with buffer instead of neuraminidase and sections incubated with neuraminidase and then stained for CA 19-9 served as controls.

*Serum assay*

Serum CA 242 levels were measured by a dissociation-enhanced lanthidine fluoroimmunoassay (DELFIA; Pharmacia Diagnostics, Uppsala, Sweden). Briefly, aliquots of patient sera were incubated for 2 h at 20°C in microwells coated with purified mouse monoclonal antibodies against the CA 50 antigen. After washes the wells were treated for 1 h with purified anti-CA 242 monoclonal antibodies complexed with europium chelate (Pharmacia). The bound europium was finally quantitated after washings by adding commercial scintillation solution (LKB/Wallac, Turku, Finland) and by counting the wells in a 1230 Arcus Fluorometer (LKB/Wallac). An upper limit of normal of 20 U $ml^{-1}$, based on serum levels of healthy blood donors, was used for the assay (Nilsson *et al.*, 1988).

### Results

*Normal pancreas*

In normal pancreas 17 out of 20 specimens (85%) stained for CA 242 (Figure 1a). A positive reaction was typically seen in the apical border of some ductal cells. Large ducts were more often positive and stained more strongly than small ducts. Normal pancreatic tissue adjacent to chronic pancreatitis or carcinoma showed the same staining pattern and intensity. Acinar cells and Langerhans' islets were consistently negative.

*Pancreatitis*

All but one specimen of chronic pancreatitis (91%) were positive for CA 242 (Figure 2). The antigen was expressed mainly in the apical border of ductal cells, but also to some extent intracellularly. The staining intensity was much stronger than in normal pancreatic ducts. Intraluminal mucus stained positively. Chronic pancreatitis was also seen in carcinoma specimens adjacent to the tumour. The staining pattern was similar to that of specimens with chronic pancreatitis only. All sections of acute pancreatitis stained for CA 242. The staining was intense and widely distributed in small terminal ducts and centro-acinar cells, whereas only some of the large ducts were positive for CA 242 (Figure 3). Thus, in most cases, the staining pattern clearly differed from that seen in chronic pancreatitis and in normal pancreata, in which centroacinar cells were negative. Acinar structures and Langerhans' islets were always negative for CA 242.

*Well to moderately differentiated adenocarcinomas*

Thirty-eight out of 41 (93%) tumours expressed CA 242 (Figure 4). Frequently, the staining was focal. In well differentiated areas, the antigen was predominantly seen in the brush border and in the mucus, which stained intensely. The staining was more diffuse and mainly intracytoplasmic in moderately differentiated areas. In many specimens with intense staining, the surrounding matrix was diffusely positive. In all cases the staining intensity of the carcinoma was stronger than that of adjacent pancreatic tissue. All eight specimens from metastases were positive and had the same staining pattern as primary tumours. No differences between

**Figure 1** Normal pancreas. Immunoperoxidase staining with the C 242 antibody (**a**), the 1116 NS 19-9 antibody (**b**) and the C 50 antibody (**c**), counterstained with haematoxylin. Bar = 100 μm. Positivity for CA 242, CA 19-9 and CA 50 was seen in some ductal cells (large arrow). Centro-acinar cells also stained for CA 50 (small arrow).

**Figure 2** Chronic pancreatitis. Immunoperoxidase staining with the C 242 antibody, counterstained with haematoxylin. Bar = 100 μm. Positivity for CA 242 is seen in ductal cells.

**Figure 3** Acute pancreatitis. Immunoperoxidase staining with the C 242 antibody, counterstained with haematoxylin. Bar = 100 μm. Small terminal ducts and centro-acinar cells stain for CA 242.

**Figure 4** Well differentiated ductal adenocarcinoma of the pancreas. Immunoperoxidase staining with the C 242 antibody, counterstained with haematoxylin. Bar = 100 μm. Malignant epithelial cells and intraluminal mucus stain intensely for CA 242.

CA 242 positive and negative carcinoma specimens could be seen using conventional histochemistry.

*Poorly differentiated and anaplastic carcinomas*

The expression of CA 242 was rather similar in poorly differentiated and anaplastic carcinomas. Two out of seven poorly differentiated adenocarcinomas and one of five anaplastic carcinomas expressed the antigen. In poorly differentiated adenocarcinomas the staining was intracytoplasmic, and the number of positive cells was smaller than in well differentiated carcinomas, and in anaplastic carcinomas cells were stained only occasionally (Figure 5). One of three metastases was positive.

*Cystic tumours*

All four mucinous cystadenomas and three cystadenocarcinomas were positive for CA 242 (Figures 6 and 7). Predominantly the apical parts of the epithelial cells and the mucus were stained, but particularly in cystadenocarcinomas intracytoplasmic staining was also seen in many cells. The staining pattern was the same as in the ductal adenocarcinomas, but more intense. Three serous cystadenomas were negative for CA 242.

*Islet cell tumours*

Five benign islet cell tumours were negative for CA 50, whereas in two of four islet cell carcinomas, the cytoplasm of a few cells stained (Figure 8).

*Sensitivity to treatment with neuraminidase*

Incubation of carcinoma specimens with 0.03 U $ml^{-1}$ neuraminidase slightly weakened the staining intensity, whereas 0.1 and 0.3 U $ml^{-1}$ totally abolished the CA 242 staining. In the control series the CA 19-9 staining was abolished by 0.3 U $ml^{-1}$ neuraminidase.

*Comparison of CA 242 with CA 19-9 and CA 50*

The staining pattern of CA 242 very much resembled that of CA 19-9, whereas a clear difference was seen between the expression of CA 242 and CA 50. In normal pancreas only some of the small ducts were positive for CA 242, whereas the CA 50 staining was uniformly distributed in small ducts and centro-acinar cells (Figure 1a–c). There was a similar difference in chronic pancreatitis. In acute pancreatitis the distribution of centro-acinar cells positive for CA 50 was uniform, whereas the staining pattern for CA 242 was more

**Figure 5** Anaplastic carcinoma of the pancreas. Immunoperoxidase staining with the C 242 antibody, counterstained with haematoxylin. Bar = 100 μm. Occasional carcinoma cells stain for CA 242 (arrows).

**Figure 6** Mucinous cystadenoma of the pancreas. Immunoperoxidase staining with the C 242 antibody, counterstained with haematoxylin. Bar = 100 μm. Predominantly apical parts but also intracytoplasmic structures of mucinous epithelial cells stain for CA 242.

patchy. In carcinomas and cystadenocarcinomas the same structures stained for CA 242, CA 50 and CA 19-9. However, the intensity of the CA 242 and CA 19-9 staining was stronger and more widely distributed in most specimens than that of CA 50. Carcinomas stained more strongly for CA 242, as for CA 19-9, than pancreatic tissue adjacent to the tumour, whereas the opposite was the case for CA 50.

Most specimens, 74 out of 113, were positive for all three antigens, whereas seven specimens were negative for all (Table I). All specimens of normal pancreas, as well as acute and chronic pancreatitis, stained for CA 50. One specimen of normal pancreas was negative for CA 242, three specimens for CA 19-9 and two specimens for both CA 242 and CA 19-9. One specimen of acute pancreatitis was negative for

**Figure 7** Cystadenocarcinoma of the pancreas. Immunoperoxidase staining with the C 242 antibody, counterstained with haematoxylin. Bar = 100 μm. The cystadenocarcinoma epithelium stains intensely for CA 242.

**Figure 8** Islet cell carcinoma. Immunoperoxidase staining with the C 242 antibody, counterstained with haematoxylin. Bar = 100 μm. A few cells stain for CA 242 (arrow).

**Table I** Comparison of the immunohistochemical expression of CA 242, CA 50 and CA 19-9 in benign and malignant pancreatic lesions

| | | *Number of specimens* | | | | | | | |
|---|---|---|---|---|---|---|---|---|---|
| *Specimens* | *Total* | *CA 242 + CA 50 + CA 19-9 +* | *CA 242 + CA 50 + CA 19-9 −* | *CA 242 + CA 50 − CA 19-9 +* | *CA 242 + CA 50 − CA 19-9 −* | *CA 242 − CA 50 + CA 19-9 +* | *CA 242 − CA 50 + CA 19-9 −* | *CA 242 − CA 50 − CA 19-9 +* | *CA 242 − CA 50 − CA 19-9 −* |
| Normal pancreas | 20 | 14 | 3 | – | – | 1 | 2 | – | – |
| Acute pancreatitis | 10 | 9 | 1 | – | – | – | – | – | – |
| Chronic pancreatitis | 11 | 10 | – | – | – | – | 1 | – | – |
| Well differentiated adenocarcinoma | 41 | 31 | 5 | 2 | – | 1 | 1 | 1 | – |
| Poorly differentiated and anaplastic carcinoma | 12 | 3 | – | – | – | 3 | 3 | 1 | 2 |
| Cystic tumours | | | | | | | | | |
| serous | 3 | – | – | – | – | – | 3 | – | – |
| mucinous | 4 | 4 | – | – | – | – | – | – | – |
| Islet cell tumours | 3 | 3 | – | – | – | – | – | – | – |
| benign | 5 | – | – | – | – | – | – | – | 5 |
| malignant | 4 | – | 2 | – | – | – | 2 | – | – |
| Total | 113 | 74 | 11 | 2 | – | 5 | 12 | 2 | 7 |

CA 19-9, and one specimen of chronic pancreatitis for both CA 19-9 and CA 242. Five well to moderately differentiated ductal adenocarcinomas, which were CA 19-9 negative, stained positively for CA 242 (Table I). On the other hand, two well to moderately differentiated and four poorly differentiated or anaplastic carcinomas were negative for CA 242, in spite of a positive CA 19-9 staining. Two CA 50-negative well differentiated carcinomas were positive for CA 242, whereas two well to moderately differentiated and six poorly differentiated or anaplastic carcinomas were CA 50 positive but CA 242 negative (Table I). Benign and malignant mucinous tumours stained for all three antigens. Serous cystadenomas were positive only for CA 50, but were negative for CA 242 and CA 19-9. Benign islet cell tumours were always negative. In islet cell carcinomas occasional cells stained for CA 242, but the number of positive cells was smaller than for CA 50, whereas these tumours were negative for CA 19-9.

*Correlation between tissue staining and serum concentration of CA 242*

Serum was available in 23 of the patients with pancreatic cancer. A value higher than 20 U $ml^{-1}$ was found in 65% of these patients (Table II). There was no clearcut correlation between the histological expression and the serum levels of the antigens.

### Discussion

Lindholm *et al.* (1983, 1985) have raised several antibodies to colorectal carcinoma cell line COLO 205. Initially one of them, the C 50 antibody, was more widely studied, and a tumour marker test, CA 50, based on this antibody has been developed (Holmgren *et al.*, 1984). The antigenic epitope of CA 50 is similar, although not identical, to that of tumour marker CA19-9 (Månsson *et al.*, 1985; Nilsson *et al.*, 1985). Monoclonal antibody C 242 was raised against the same carcinoma cell line as CA 50 (Lindholm *et al.*, 1985). The exact nature of the antigenic determinant of CA 242 is not known, but it seems to be a sialylated carbohydrate structure related to type I chain (O. Nilsson, personal communication). Thus, chemically it would be closely related to CA 19-9 and CA 50, which is further supported by the similarities in immunohistochemical expression of these three markers. The sensitivity of the CA 242 antigen to neuraminidase implies that sialic acid is an essential part of the structure.

**Table II** CA 242 in tissue and serum of patients with pancreatic cancer

| Specimens | Patient number | CA 242 Tissue[a] | CA 242 Serum[b] |
|---|---|---|---|
| Small, well to moderately differentiated ductal adenocarcinomas | 1 | + + | 126 |
| | 2 | + + | 76 |
| | 3 | + + | 15 |
| | 4 | + | 9 |
| | 5 | + | 5 |
| | 6 | + + | 5 |
| Large, well to moderately differentiated ductal adenocarcinomas | 7 | + | 1960 |
| | 8 | + + | 690 |
| | 9 | + | 690 |
| | 10 | + | 219 |
| | 11 | + | 153 |
| | 12 | + | 135 |
| | 13 | + | 9 |
| | 14 | – | 5 |
| Poorly differentiated and anaplastic carcinomas | 15 | + + | 670 |
| | 16 | – | 207 |
| | 17 | + | 47 |
| | 18 | + | 5 |
| Cystadenocarcinomas | 19 | + + | 2320 |
| | 20 | + | 910 |
| | 21 | + | 470 |
| Islet cell carcinomas | 22 | + | 24 |
| | 23 | – | 5 |

[a]Arbitrary scoring, wide or uniform distribution of CA 242 was scored as + +, positivity of only a few glands or occasional cells as +. The intensity of the staining affected scoring only in borderline cases. [b]Concentration in units $ml^{-1}$, cut-off level 20 U $ml^{-1}$.

We have previously reported the tissue expression of CA 19-9 and CA 50 in pancreatic lesions (Haglund *et al.*, 1986 *a, b*). The CA 242 antigen is, like CA 19-9 and CA 50, easily demonstrated by immunoperoxidase technique in formalin-fixed, paraffin-embedded specimens. Although CA 242 is apparently a normal constituent of the human pancreas, it was very weakly expressed in healthy tissue. On the other hand, it was strongly expressed in most carcinomas, and always much stronger than in adjacent normal pancreas. In acute pancreatitis the expression was much stronger than in normal pancreas and centro-acinar cells also stained, which was not the case in normal pancreas or in chronic pancreatitis. The staining of CA 242 resembled that of CA 19-9, whereas the expression of CA 50 was stronger and more widely distributed in benign pancreatic tissue than that of CA 242 and CA 19-9. The serum levels of the three tumour markers are low in healthy individuals (DelVillano *et al.*, 1983; Holmgren *et al.*, 1984; Nilsson *et al.*, 1988), yet the tissue expression of CA 50 in normal pancreas is quite strong. Therefore, it is postulated that these antigens are not usually shed into the blood stream from normal pancreatic tissue. In pancreatitis about 1/5 of the patients have slightly elevated serum levels of CA 19-9 and CA 50 (Haglund *et al.*, 1986*c*, 1987), whereas, in a preliminary study, no elevated levels of CA 242 have been found (Kuusela *et al.*, submitted). The differences in tissue and serum expression in pancreatitis show that these three markers are distinctly different.

CA 19-9, being a sialylated Lewis[a] structure, cannot be expressed by Lewis negative individuals. An interesting question is whether CA 242, like CA 50 (Haglund *et al.*, 1986*b*), is expressed in Lewis negative specimens. Previously the expression of Lewis[a] and Lewis[b] in CA 19-9 negative specimens has been reported (Haglund *et al.*, 1986*a*). Five well to moderately differentiated ductal adenocarcinomas, which were negative for CA 19-9, stained positively for CA 242. Three of these were Lewis[b] positive, one Lewis[a] and Lewis[b] positive, and only one Lewis negative. Five other Lewis negative specimens were both CA 19-9 and CA 242 negative. Thus, it seems that Lewis negative individuals may express CA 242 weakly. On the other hand, these were all poorly differentiated or anaplastic carcinomas, which seldom and weakly express CA 242. The expression of CA 242 in pancreatic carcinomas seems to be more dependent on the degree of differentiation than the expression of CA 19-9 and CA 50 (Haglund *et al.*, 1986*a, b*).

Although most carcinoma specimens were positive or negative for all three antigens, 11 specimens stained for either CA 242 or CA 19-9 and 10 specimens for either CA 242 or CA 50. This also supports previous findings that the C 242 antibody detects a different antigenic determinant from the 19-9 and C 50 antibodies. Notably, no specimen was positive for CA 242 but negative for both CA 19-9 and CA 50.

The expression of CA 242 correlates with the degree of differentiation in the ductal carcinomas, being strongest in well differentiated tumours. The findings are in concordance with those for CA 19-9 and CA 50 (Haglund *et al.*, 1986*a, b*; Ichihara *et al.*, 1988). The sequential change in antigenic distribution from normal epithelium to poorly differentiated and anaplastic carcinomas represents loss of polar distribution of membrane associated antigens, which has been described in various gastrointestinal carcinomas (Ahnen *et al.*, 1982; Nagura *et al.*, 1983; Ichihara *et al.*, 1988). However, the differences between the staining pattern in benign and malignant pancreatic lesions are not clear enough to make immunohistochemical staining of CA 242 useful in distinguishing between chronic pancreatitis and carcinoma. Similar results have previously been reported for normal and neoplastic colon mucosa (Ouyang *et al.*, 1987). The expression of CA 242 in normal and neoplastic epithelium of other gastrointestinal organs is still poorly known. However, due to the strong expression in many pancreatic carcinomas, compared to adjacent pancreatic tissue, C 242 might be useful for immunolocalisation.

There was no correlation between the tissue expression and the serum levels of CA 242, which is in confirmity with previous findings for CA 19-9 and CA 50 (Haglund *et al.*, 1986*a*, *b*; Nishida *et al.*, 1988). Many tissue positive carcinomas were associated with a normal or only slightly elevated serum level of CA 242. On the other hand, high serum values were seen in patients with a weakly or moderately positive staining, and in one patient even in spite of a negative tissue staining. This indicates that factors other than antigen production in the tumour affect the serum levels. These factors may include invasion, extent and localisation of tumour spread as well as factors affecting the metabolism and excretion of the antigen.

In clinical practice, CA 19-9 and CA 50 have been found to be useful tumour markers in the diagnosis and follow-up of gastrointestinal tumours, particularly pancreatic cancer (Ritts *et al.*, 1984; Jalanko *et al.*, 1984; Haglund *et al.*, 1986*c*, 1987). Both CA 19-9 and CA 50 are better tumour markers in the diagnosis of pancreatic cancer than previously available markers, e.g. CEA (Haglund *et al.*, 1986*a*; Kuusela *et al.*, 1987). However, the disadvantage of these markers is that elevated values are found also in association with certain benign diseases, sometimes in chronic pancreatitis and especially in benign extrahepatic biliary obstruction. In this study we have shown that pancreatic carcinomas also strongly express CA 242, an antigen closely related to, but still different from, CA 50 and CA 19-9. This new tumour associated antigen seems potentially useful as a tumour marker in the diagnosis of pancreatic cancer. However, further studies on the serum expression of CA 242 in patients with pancreatic cancer and in relevant benign controls are needed to evaluate whether the serum assay for CA 242, alone or combined with other tests, has any advantages over CA 19-9 and CA 50.

The authors thank Dr L. Lindholm and Dr H. Koprowski for kindly supplying antibodies. This study was supported by grants from Finska Läkaresällskapet, the Finnish Cancer Society and the Stena Foundation.

### References

AHNEN, D.J., NAKANE, P.K. & BROWN, W.R. (1982). Ultrastructural localization of carcinoembryonic antigen in normal intestine and colon cancer: abnormal distribution of CEA on the surface of colon cancer cells. *Cancer*, **49**, 2077.

DEL VILLANO, B.C., BRENNAN, S., BROCK, P. & 8 others (1983). Radioimmunometric assay for a monoclonal antibody-defined tumor marker, CA 19-9. *Clin. Chem.*, **29**, 549.

HAGLUND, C., KUUSELA, P., JALANKO, H. & ROBERTS, P.J. (1987). Serum CA 50 as a tumor marker in pancreatic cancer: a comparison with CA 19-9. *Int. J. Cancer*, **39**, 477.

HAGLUND, C., LINDGREN, J., ROBERTS, P.J. & NORDLING, S. (1986*a*) Gastrointestinal cancer associated antigen CA 19-9 in histological specimens of pancreatic tumours and pancreatitis. *Br. J. Cancer*, **53**, 189.

HAGLUND, C., LINDGREN, J., ROBERTS, P.J. & NORDLING, S. (1986*b*). Tissue expression of the tumor marker CA 50 in benign and malignant pancreatic lesions. A comparison with CA 19-9. *Int. J. Cancer*, **38**, 841.

HAGLUND, C., ROBERTS, P.J., KUUSELA, P., SCHEININ, T.M., MÄKELÄ, O. & JALANKO, H. (1986*c*). Evaluation of CA 19-9 as a serum tumour marker in pancreatic cancer. *Br. J. Cancer*, **53**, 197.

HOLMGREN, J., LINDHOLM, L., PERSSON, B. & 8 others (1984). Detection by monoclonal antibody of carbohydrate antigen CA 50 in serum of patients with carcinoma. *Br. Med. J.*, **288**, 1479.

ICHIHARA, T., NAGURA, H., NAKAO, A., SAKAMOTO, J., WATANABE, T. & TAKAGI, H. (1988). Immunohistochemical localization of CA 19-9 and CEA in pancreatic carcinoma and associated diseases. *Cancer*, **61**, 324.

JALANKO, H., KUUSELA, P., ROBERTS, P., SIPPONEN, P., HAGLUND, C. & MÄKELÄ, O. (1984). Comparison of a new tumour marker, CA 19-9$^{TM}$, with alpha-fetoprotein and carcinoembryonic antigen in patients with upper gastrointestinal diseases. *J. Clin. Pathol*, **37**, 218.

KOPROWSKI, H., HERLYN, M., STEPLEWSKI, Z. & 4 others (1979). Colorectal carcinoma antigens detected by hybridoma antibodies. *Somat. Cell Genet.*, **5**, 957.

KUUSELA, P., HAGLUND, C., ROBERTS, P.J. & JALANKO, H. (1987). Comparison of CA-50, a new tumour marker, with carcinoembryonic antigen (CEA) and alpha-fetoprotein (AFP) in patients with gastrointestinal diseases. *Br. J. Cancer*, **55**, 673.

LINDHOLM, L., HOLMGREN, J., SVENNERHOLM, L. & 5 others (1983). Monoclonal antibodies against gastrointestinal tumour-associated antigens isolated as monosialogangliosides. *Int. Arch. Allergy Appl. Immunol.*, **71**, 178.

LINDHOLM, L., JOHANSSON, C., JANSSON, E.-L., HALLBERG, C. & NILSSON, O. (1985). An immunoradiometric assay (IRMA) for the CA-50 antigen. In *Tumour Marker Antigen*, Holmgren, J. (ed.) p. 123. Studentlitteratur: Lund, Sweden.

MAGNANI, J.L., NILSSON, B., BROCKHAUS, M. & 4 others (1982). A monoclonal antibody-defined antigen associated with gastrointestinal cancer is a ganglioside containing sialylated lacto-N-fucopentaose II. *J. Biol. Chem.*, **257**, 14365.

MÅNSSON, J.E., FREDMAN, P., NILSSON, O., LINDHOLM, L., HOLMGREN, J. & SVENNERHOLM, L. (1985). Chemical structure of carcinoma ganglioside antigens defined by monoclonal antibody C-50 and some allied gangliosides of human pancreatic adenocarcoma. *Biochim. Biophys. Acta*, **834**, 110.

NAGURA, H., TSUTSUMI, Y., SHIODA, Y. & WATANABE, K. (1983). Immunohistochemistry of gastric carcinomas and associated diseases: novel distribution of carcinoembryonic antigen and secretory component on the surface of gastric cancer cells. *J. Histochem. Cytochem.*, **31**, 193.

NILSSON, O., MÅNSSON, J.-E., LINDHOLM, L., HOLMGREN, J. & SVENNERHOLM, L. (1985). Sialosyllactotetraosylceramide, a novel ganglioside antigen detected in human carcinomas by a monoclonal antibody. *FEBS Lett.*, **182**, 398.

NILSSON, O., JANSSON, E.-L., JOHANSSON, C. & LINDHOLM, L. (1988). CA-242, a novel tumor-associated carbohydrate antigen with increased tumour specificity and sensitivity. *J. Tumor Marker Oncol.*, **3**, 314.

NISHIDA, K., MIYAGAWA, H., YOSHIKAWA, T. & KONDO, M. (1988). Concentration and localization of carbohydrate antigen 19-9 in tissues of pancreatic cancer. *Oncology*, **45**, 166.

OUYANG, Q., VILIEN, M., RAVN JUHL, B., GRUPE LARSEN, L. & BINDER, V. (1987). CEA and carbohydrate antigens in normal and neoplastic colon mucosa. *Acta Pathol. Microbiol. Immunol. Scand. A*, **95**, 177.

RITTS, R.E. Jr., DEL VILLANO, B.C., GO, V.L.W., HERBERMAN, R.B., KLUG, T.L. & ZURAWSKI, V.R. Jr. (1984). Initial clinical evaluation of an immunoradiometric assay for CA 19-9 using the NCI serum bank. *Int. J. Cancer*, **33**, 339.